# Quantification of pituitary 5-HT_1B_ receptors with positron emission tomography: Negligible specific binding despite conspicuous uptake

**DOI:** 10.1177/0271678X261417194

**Published:** 2026-02-08

**Authors:** Martin Gärde, Katarina Varnäs, Johan Lundberg, Jonas Svensson, Lars Farde, Granville J Matheson, Mikael Tiger

**Affiliations:** Centre for Psychiatry Research, Department of Clinical Neuroscience, Karolinska Institutet & Stockholm Health Care Services, Region Stockholm, Sweden

**Keywords:** [^11^C]AZ10419369, 5-HT_1B_ receptor, blood-brain barrier, kinetic modeling, pituitary

## Abstract

While Positron Emission Tomography (PET) images with the 5-HT_1B_ receptor specific radioligand [^11^C]AZ10419369 show pronounced uptake in the pituitary region, and experimental studies support 5-HT_1B_ receptor involvement in pituitary hormonal release, this uptake remains unquantified. In the present study we applied invasive and non-invasive models to evaluate pituitary 5-HT_1B_ receptor binding. Ten subjects underwent PET with [^11^C]AZ10419369, of which six participated in three additional PET-examinations after pretreatment with increasing doses of the 5-HT_1B_ receptor antagonist AZD3783. While [^11^C]AZ10419369 binding in the brain displayed dose-dependent reductions after AZD3783, no dose-dependent inhibition of binding was observed for the pituitary. Distribution volume ratios were plotted against occupancy values to graphically estimate regional differences in non-displaceable binding, thereby allowing for estimation of *BP*_ND_ in extracerebral regions. Using this method, baseline pituitary *BP*_ND_ appears to be negligible, implying that most of the pituitary [^11^C]AZ10419369 uptake is comprised of free or nonspecifically bound radioligand. Our findings highlight potential pitfalls when assuming that conspicuous regional radioligand uptake indicates presence of specific binding.

## Introduction

The serotonin 1B (5-HT_1B_) receptor has been implicated in the pathophysiology of mood disorders^1^ and migraine,^2^ that is, conditions both featuring disturbances in pituitary hormone signaling.^3–6^ 5-HT_1B_ receptor involvement in pituitary gland hormone release is supported by experimental data: 5-HT_1B_ receptor agonists increase growth hormone (GH) concentration in rat pituitary tissue^7^ and raise serum concentrations of GH and prolactin (PRL) in vivo in humans.^8,9^

**Figure 1.**
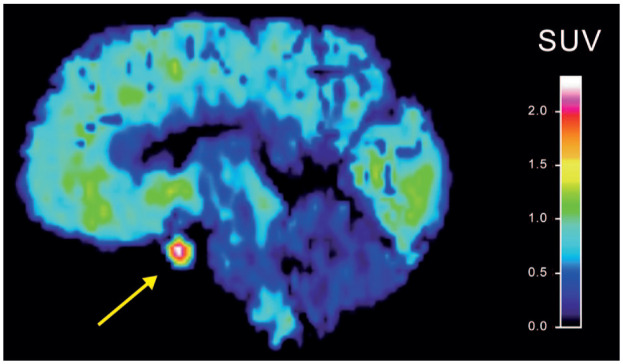
SUV image of a representative baseline PET examination showing high [^11^C]AZ10419369 uptake in the pituitary region.

Human 5-HT_1B_ receptors can be quantified in vivo using positron emission tomography (PET) and 5-HT_1B_ receptor-specific radioligands such as [^11^C]AZ10419369^10^ or [^11^C]P943.^11^ Both [^11^C]P943^12^ and [^11^C]AZ10419369 (Figure 1) exhibit conspicuously high uptake in the pituitary region. Nevertheless, pituitary 5-HT_1B_ receptor density or binding has not been characterized in humans. The lack of pituitary autoradiography data may be explained by the difficulty involved in removing pituitary tissue from the surrounding sella turcica during standard brain autopsy.

The degree to which the high uptake in the pituitary region reflects specific binding can be assessed by performing PET before and after administration of a competing 5-HT_1B_ receptor ligand. The change in signal is then used to estimate the proportion of occupied receptors,^13^ an approach referred to as an occupancy study. AZD3783 is a high affinity 5-HT_1B_ receptor antagonist which displays dose-dependent 5-HT_1B_ receptor occupancy both in vitro and in vivo,^14,15^ making it suitable for this purpose.

The pituitary gland lies outside the blood-brain barrier (BBB),^16,17^ implying that the concentration of pituitary parent [^11^C]AZ10419369 and its radioactive metabolites may differ from the concentration in brain. In this regard, [^11^C]AZ10419369 is well suited for pituitary quantification since it displays negligible formation of radioactive metabolites during a PET examination.^10,18^ Non-invasive quantification methods avoid arterial cannulation and cumbersome plasma sampling but require a reference tissue with a non-displaceable volume of distribution (*V*_ND_) identical to that of the target region. Although [^11^C]AZ10419369 has been validated for non-invasive quantification of 5-HT_1B_ receptors using the cerebellum as reference,^10^ the pituitary gland largely consists of glandular tissue, while the cerebellum is comprised of neuronal tissue, making the assumption of identical *V*_ND_ tenuous. Nevertheless, the cerebellum has proven successful as reference region in measurement of pituitary dopamine D_2_ receptor occupancy,^19^ warranting assessment of its use as a normalizing factor in pituitary 5-HT_1B_ receptor quantification.

The aim of this study was to quantify pituitary 5-HT_1B_ receptor binding in healthy volunteers examined with [^11^C]AZ10419369 before and after pretreatment with different doses of AZD3783. Several methods were applied. The specificity of pituitary binding was assessed in terms of changes in distribution volume (*V*_T_) and non-displaceable binding potential (*BP*_ND_) before and after AZD3783 administration. Changes in pituitary *BP*_ND_ and *V*_T_ after AZD3783 pretreatment were compared to the corresponding *BP*_ND_ and *V*_T_ changes in brain regions of interest. An estimate of pituitary specific binding adjusted for regional variation in *V*_ND_ was obtained based on the relationship between brain 5-HT_1B_ receptor occupancy and distribution volume ratios (DVRs) calculated for the pituitary relative to the cerebellum at different doses of AZD3783.

## Materials and methods

### Subjects and study design

The data used in this study were drawn from previous projects investigating brain [^11^C]AZ10419369 binding before and after AZD3783 administration^10,15^

This study received approval from the Medical Products Agency, the Radiation Safety Committee, and the Ethical Review Board in Stockholm reference number 2006/331-31/2 and 2007/89-31/2. The study was performed in accordance with ICH/Good Clinical Practice, AstraZeneca policy of bioethics and the ethical principles described in the 1964 Declaration of Helsinki. Informed consent was obtained from all subjects prior to study initiation.

Ten healthy men aged 21–34 years underwent a baseline PET with [^11^C]AZ10419369. Subsequently, in six of these subjects (subjects 1–6) three additional PET exams with [^11^C]AZ10419369 were performed after an oral dose of AZD3783. The AZD3783 dose was gradually increased at each occasion.

T1-weighted Magnetic Resonance Imaging (MRI) was performed using a 1.5T General Electrics Signa (GE, Milwaukee, WI, USA) system. The Siemens ECAT EXACT HR PET system was used to measure radioactivity in a 93 min series of consecutive time frames (9 × 20s, 3 × 60s, 3 × 180s and 13 × 360s frames). [^11^C]AZ10419369 was prepared as previously described.^10^ An arterial input function was collected using automated arterial blood sampling for 5 min followed by manual samples drawn at each frame. Injected radioactivity, molar activity, and injected mass for each PET, are available in the supplement (Table s1), alongside PET data reconstruction parameters. See original publications for further details.^10,15^

### Image analysis

#### Regions of interest

The following brain regions of interest (ROIs) employed in the analysis were chosen based on their moderate to high 5-HT_1B_ receptor binding in the literature^10,20–23^: Anterior Cingulate Cortex (ACC), Occipital Cortex (OC), Orbitofrontal Cortex (OFC), and the dorsal and ventral Striatum (STR). Cerebral gray matter (GM) was used to calculate brain occupancy in the graphical estimation of specific pituitary 5-HT_1B_ receptor binding. All brain ROIs were defined on MRI images employing the automated FreeSurfer analysis suite (version 6, http://surfer.nmr.mgh.harvard.edu/).

PET images were motion corrected using a frame-by-frame rigid-body registration.^24^ T1-weighted MRI images were co-registered to a time-weighted summated PET-image using Statistical Parametric Mapping version 12 (SPM12, Wellcome Department of Cognitive Neurology, University College, London, UK). Regional time-activity curves (TACs) were derived by using the resulting co-registration matrix to project ROIs onto the realigned dynamic PET-image.

Given negligible cerebellar 5-HT_1B_ receptor binding,^25^ a modified^26^ FreeSurfer cerebellar ROI was used as reference region. Because the FreeSurfer suite lacks automated delineation of the pituitary gland, the pituitary ROI was delineated using the following approach. First, using the MR data a small cuboid mask, reliably containing the pituitary gland, was produced by using the landmarks of the optic chiasm, the most ventral part of the frontal cortex, and the AC-plane derived from the FreeSurfer labels. Using the previously obtained co-registration matrix, this mask was moved into individual PET-space of the baseline examination, where a version of a previously described automated method^27^ identified the most intense voxel within the mask. In an iterative process, neighboring voxels were added by order of highest count to ensure a contiguous ROI mask. The process was terminated once the mask reached the predefined size of 420 mm^3^, corresponding to the mean pituitary volume in the age group of our subjects.^28^

#### Quantification of [^11^C]AZ10419369 binding

Distribution volumes (*V*_T_) were calculated using the one tissue compartment model (1-TCM) with the plasma radioactivity concentration of [^11^C]AZ10419369 as input function.^29^ Two-tissue compartment (2-TC) estimation of *V*_T_ and rate constants was omitted as the 1-TCM has been shown to be sufficient for describing [^11^C]AZ10419369 kinetics.^10^ Observations of high pituitary blood flow in animals,^30,31^ and the positive correlation between cerebral blood flow (CBF) and cerebral blood volume (CBV) found in humans,^32^ suggest that the blood volume fraction (*V*_B_) of the pituitary may be substantially larger than that of the brain. As kinetic models may produce biased results if not properly corrected for tissue blood volumes,^33,34^ distribution volumes were estimated with *V*_B_ as a free parameter.

*BP*_ND_ based on the cerebellum ROI as a reference region was calculated employing the simplified reference tissue model (SRTM).^35^ To compensate for the putative bias from regional variations in *V*_B_, we also applied a modified version of the simplified reference tissue model (SRTM-V)^36^ with the pituitary *V*_B_ set as a free parameter fitted by non-linear least squares estimation. Reference tissue *V*_B_ was set to the mean cerebellar *V*_B_ estimated with 1-TCM.

#### Occupancy calculations

Receptor occupancy (%) was calculated from the obtained brain regional *BP*_ND_ values using the following equation:

(1) $$Occupancy=\left(1-\frac{BP_{ND\left(drug\right)}}{BP_{ND\left(baseline\right)}}\right)\times 100$$

Where *BP*_ND drug_ and *BP*_ND baseline_ refers to *BP*_ND_ values estimated after and before AZD3783 administration respectively.

#### Graphical estimation of *BP*_ND_ from DVRs and gray matter occupancy

There is substantial inter- and intra-subject variation in [^11^C]AZ10419369 brain *V*_T_, which has been suspected to result from variability in [^11^C]AZ10419369 plasma protein binding.^10^ This variation was also apparent for the pituitary. Distribution volume ratios (DVRs), obtained by normalizing the target region distribution volume to the distribution volume of a reference region within the brain, avoid the issue of variability in radioligand protein binding. However, bias may still be introduced by differences in target and reference region non-displaceable binding (i.e. *V*_ND_) which may be problematic with regards to the pituitary ROI. We herein propose a graphical method to estimate discrepancies in reference and target *V*_ND_ based on the relationship between DVR and brain occupancy.

For any ROI, the relationship between distribution volumes and *BP*_ND_ may be described as follows:

(2) $$V_{T}=V_{ND}\left(BP_{ND}+1\right)$$

Assuming negligible *BP*_ND_ in the reference region, integration of DVR in equation (2) can be expressed as:

(3) $$DVR_{\left(T/R\right)}=\frac{V_{ND\left(T\right)}}{V_{T\left(R\right)}}\left(BP_{ND}+1\right)$$

Where *V*_ND(T)_ and *V*_T(R)_ denote the non-displaceable distribution volumes of the target and the distribution volume of the reference region, respectively. Because [^11^C]AZ10419369 has negligible specific binding in the cerebellum ROI employed as reference, *V*_T(R)_ is in the following represented by *V*_ND(R)_. Variation in occupancy can be accounted for by the replacement of *BP*_ND_ with *BP*_ND Baseline_ * (1 − *O*) where *O* is the fractional receptor occupancy:

(4) $$DVR=\frac{V_{ND\left(T\right)}}{V_{ND\left(R\right)}}\left(1+BP_{ND\;Baseline}\left(1-O\right)\right)$$

In this evaluation, *O* has been calculated from the cerebral gray matter *BP*_ND._ Assuming 100% occupancy can be reached at high doses of competitor drug, a plot of *x* = 1 − *O* and *y* = DVR produces a linear relationship with the y-intercept equal to the ratio between the target and reference region non-displaceable distribution volumes (*V*_ND(T)_/*V*_ND(R)_). In such a plot, the *V*_ND(T)_/*V*_ND(R)_ ratio can be estimated using least squares regression and *BP*_ND Baseline_ can then be obtained from the slope of the line that is, *V*_ND(T)_/*V*_ND(R)_ × *BP*_ND Baseline_. In this way, if the assumption of equal *V*_ND_ in both the target and reference region holds, then the y-intercept should be equal to 1. If it is higher or lower than 1, then it implies that the target region has either higher or lower *V*_ND_ than the reference region respectively. Another requirement of this approach is equal free passage across the BBB for the competitor drug. This assumption appears reasonable given the close agreement between the unbound plasma concentration required for 50% occupancy *K*_i,plasma_ in vivo^15^ and in vitro^14^ of AZD3783.

#### Statistical analysis

Invasive and reference model outcomes were correlated using Pearson’s correlation coefficient.

Statistical analysis and kinetic modeling were performed using kinfitr^37^ in R, version 4.2.1 “Funny-Looking Kid.”^38^

## Results

Subject 10 was excluded from analysis due to an aberrant shape of the blood and plasma curves suspected to have resulted from extravasation of injected radioligand. Due to technical problems,^10,15^ data were not available for subject 3 at the 40 mg dose or subject 6 at the 4 mg and 20 mg doses of AZD3783. The fraction of unchanged [^11^C]AZ10419369 at the end of PET was ⩾97% across subjects.

### Regional [^11^C]AZ10419369 binding and displacement after AZD3783 pretreatment

**Figure 2.**
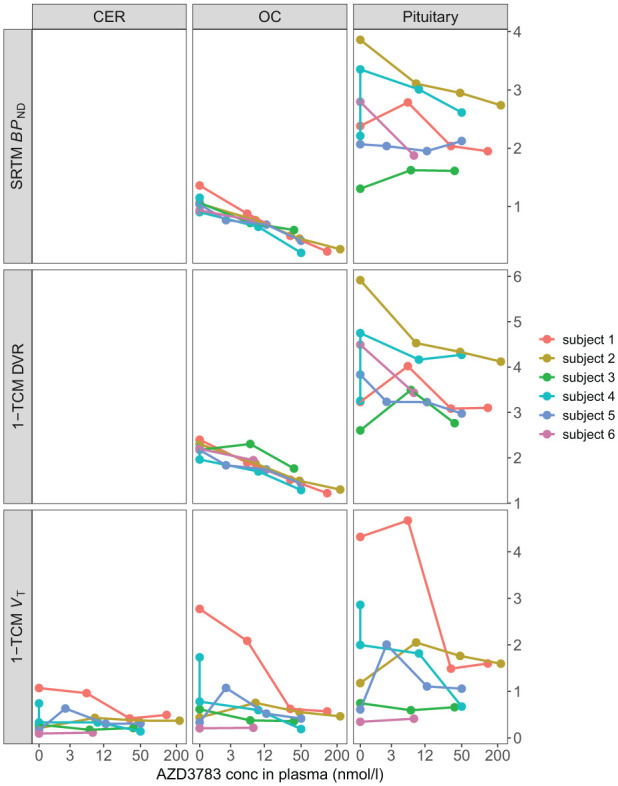
Regional outcome measures at different doses of AZD3783. SRTM *BP*_ND_: Binding potential non-displaceable calculated using SRTM; 1-TCM DVR: 1-TCM derived distribution volume ratio; 1-TCM *V*_T_: 1-TCM derived volume of distribution; CER: Cerebellar reference region; OC: Occipital cortex. X-axis scaled to pseudo-log for readability.

**Figure 3.**
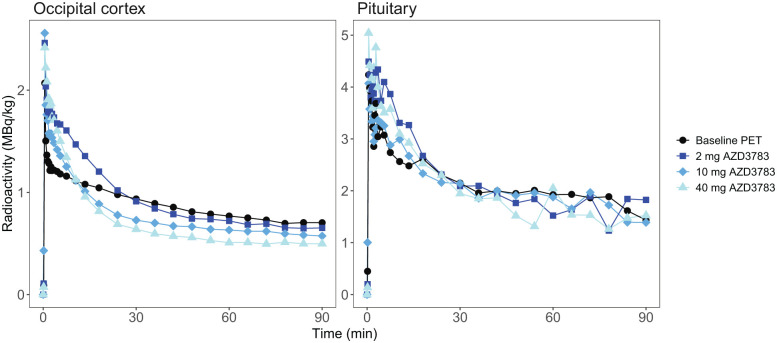
Time activity Curves (TACs) of subject 2 occipital cortex and pituitary radioactivity standardized to injected radioactivity and weight.

Baseline average pituitary [^11^C]AZ10419369 *V*_T_ estimated with 1-TCM was 1.63 ± 1.12 (mean ± SD) with mean pituitary and cerebellar *V*_B_ fitted to 13.08 and 7.39% respectively. Pituitary and cerebral ROI *V*_T_ values were highly correlated (*r* = 0.91 − 0.93, *p* < 0.001) but AZD3783 pretreatment did not dose-dependently reduce *V*_T_ within the brain or the pituitary (Figure 2). For two subjects (subject 2 and 5) *V*_T_ even increased across ROIs compared to baseline at the lowest dose of AZD3783. This was surprising given the dose-dependent reduction in brain radioactivity that was visible at equilibrium on cerebral time activity curves normalized for injected radioactivity and body weight (Figure 3). No relationship between intrasubject variation in injected mass and *V*_T_ was observed (Supplement Table s1). In the cerebellum ROI, where AZD3783 effects on 5-HT_1B_ receptor binding should be negligible, the within individual average *V*_T_ coefficient of variation (CV) was 0.44. This was notably higher than the average within individual cerebellar radioactivity normalized to injected radioactivity and weight CV of 0.12.

Baseline pituitary *BP*_ND_ estimated using SRTM and SRTM-V were 2.42 ± 0.93 and 3.27 ± 1.36 respectively which is higher than the [^11^C]AZ10419369 *BP*_ND_ previously reported for brain regions, save the pallidum.^10,21,23^ While 5-HT_1B_ *BP*_ND_ decreased dose-dependently in response to AZD3783 within the brain, no dose-dependent inhibition of pituitary 5-HT_1B_ receptor *BP*_ND_ was observed (Figure 2). Although pituitary *V*_B_ was estimated to 15.02% on average using SRTM-V, thereby violating the SRTM assumption of equal *V*_B_ in target and reference regions,^33^ *BP*_ND_ values estimated using both models were closely correlated (*r* = 0.94, *p* < 0.001) and equally unresponsive to AZD3783. Considering that dose-dependent reductions of 5-HT_1B_ receptor *BP*_ND_ were absent specifically in the pituitary ROI, we do not consider SRTM or SRTM-V *BP*_ND_ to be suitable measures of specific 5-HT_1B_ receptor binding in this structure. Pituitary occupancy calculated from SRTM *BP*_ND_ at different doses of AZD3783 are provided in the supplement (Table s2) along with the occupancy results for ROIs within the brain (Table s3).

### Graphical estimation of *V*_ND_ ratios and pituitary specific binding

**Figure 4.**
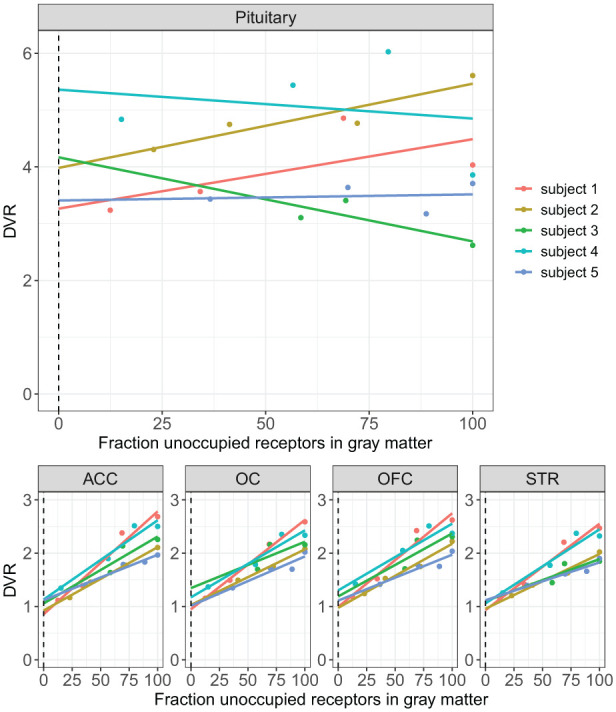
Distribution volume ratios (DVR) for subject 1–5 plotted against cerebral gray matter occupancy estimated from SRTM. ACC: Anterior cingulate cortex; OC: Occipital cortex; OFC: Orbitofrontal cortex; STR: Striatum. The dashed line marks the y-intercept which equals the DVR ratio at 100% occupancy.

The *V*_ND_ ratio was not estimated for subject 6 as the graphical analysis would have relied on only two data points. The mean *V*_ND_ ratio in the examined within-brain ROIs for the remaining five subjects was 1.02 ± 0.15 (mean ± SD) in the ACC, 1.09 ± 0.26 (mean ± SD) in the OFC, 1.10 ± 0.20 in the OC and 1.03 ± 0.10 in the STR. The mean pituitary *V*_ND_ ratio was 4.04 ± 1.03 corresponding to a mean baseline pituitary *BP*_ND_ of 0.07 ± 0.39 calculated from the slopes of the lines for each subject (Figure 4). Standard errors (SEs) for the pituitary DVR employed in the estimation of pituitary *BP*_ND_ baseline, are available in the supplement (Table s4).

## Discussion

In this PET analysis we aimed to quantify pituitary 5-HT_1B_ receptors in human subjects using the 5-HT_1B_ receptor specific radioligand [^11^C]AZ10419369. The specificity of [^11^C]AZ10419369 binding was assessed by measuring the extent to which [^11^C]AZ10419369 could be displaced by 5-HT_1B_ receptor-specific antagonist AZD3783. While all six subjects pretreated with AZD3783 displayed dose-dependent reductions in *BP*_ND_ in brain regions, no evident dose-dependent inhibition of radioligand binding was observed for the pituitary.

A critical assumption of reference tissue models is the existence of a reference region with a *V*_ND_ equal to that of the target region. At 100% occupancy, where the contribution of *V*_S_ to *V*_T_ is negligible, the DVR for any such two regions becomes a ratio of the target to reference *V*_ND_. For regions where the reference-based model assumption of uniform *V*_ND_ is valid, this ratio is therefore expected to be 1 at 100% occupancy. Indeed, in our graphical estimation of the *V*_ND_ ratio in cerebral ROIs the *V*_ND_ ratio ranged from 1.02 ± 0.15 (mean ± SD) in the ACC to 1.10 ± 0.20 in the OC corroborating the validity of the cerebellum as a reference region for [^11^C]AZ10419369 binding quantification within the brain. By contrast, the pituitary to cerebellum *V*_ND_ ratio was 4.04 ± 1.03, indicating a pituitary *V*_ND_ over four times that of the reference region. Given this large *V*_ND_ mismatch, reference tissue-based *BP*_ND_ was deemed an unsuitable outcome parameter for the pituitary.

Pituitary and cerebral ROIs differ in their composition and physiology in several respects that might have implications for their respective non-specific binding. Firstly, the pituitary ROI is largely comprised of glandular rather than neuronal tissue, meaning that the distribution volume of [^11^C]AZ10419369 non-specifically bound to cellular constituents might well be quite different from that of the brain. Secondly, although studies examining active BBB efflux of [^11^C]AZ10419369 in humans are lacking, rats, mice, and guinea pigs all display 2–3 fold increases in [^11^C]AZ10419369 brain uptake after pretreatment with ABC transporter inhibitor cyclosporin.^39^ In contrast, cyclosporin pretreatment has no significant effect on pituitary uptake of ^11^C-metoclopramide in humans,^40^ consistent with the absence of ABC-mediated efflux in this region. Active extrusion of radioligand from brain, but not pituitary, tissue would be expected to contribute to a lower cerebellar *V*_ND_ relative to that of the pituitary, leading reference-tissue methods to overestimate pituitary *BP*_ND_.

The strong correlation between *V*_T_ values for regions inside and outside the BBB may indicate variability related to the common denominator that is, blood. Cerebellar *V*_T_ showed substantially larger within-subject variability than cerebellar radioactivity normalized to weight and injected radioactivity, indicating contribution of variability from a source other than free radioligand. To our knowledge, measurements of the intrasubject variability in [^11^C]AZ10419369 *f*_p_ has not been published, however, between-subject variability has been found to be considerable, with a CV of 38%.^20^ Furthermore, the structural similarity^15^ between AZD3783 and [^11^C]AZ10419369 implies that AZD3783 could compete with radioligand plasma protein binding possibly contributing to the inconsistent effects on *V*_T_ by AZD3783 observed here. Both subjects with marked increase in *V*_T_ at first dose of antagonist also displayed a concurrent large increase (>100% increase) in K1 across regions indicative of increased blood extraction. Increases in radioligand *f*_p_ and K1 have been previously observed after administration of unlabeled tracer leading to masking of displacement of specific brain binding.^41,42^ In the present data set, the *f*_p_ of [^11^C]AZ10419369 was not measured as the method was not yet implemented locally at the time of data collection. Since we could not confirm, or correct for, the suspected variability in *f*_p_ in the present study, the use of *V*_T_ in the quantification of 5-HT_1B_ receptor binding was considered inappropriate.

After adjusting for the high pituitary-to-cerebellum *V*_ND_ ratio, mean baseline pituitary 5-HT_1B_ receptor *BP*_ND_ was 0.07 ± 0.39 (mean ± SD) compared to the unadjusted value of 2.42 ± 0.93 calculated using SRTM with the cerebellum as reference. This result agrees with simulations demonstrating that if the *V*_ND_ in the target region substantially exceeds that of the reference region, reference tissue models markedly overestimate target *BP*_ND_, particularly in regions with low specific binding.^33^

We demonstrated that plotting fractional gray matter occupancy against DVR can be used to assess regional variation in *V*_ND._ Many mathematical models used for quantification and post-quantification analysis of PET imaging data assume spatially homogeneous *V*_ND_ across the brain, however some degree of regional variation in *V*_ND_ is to be expected, and has been demonstrated even for radioligands with a validated reference region.^43^ The graphical approach outlined above can be used in testing the extent to which this assumption is met. Moreover, although we derived DVR from distribution volumes, it could just as well be obtained from reference tissue approaches, thereby eliminating the need for arterial blood sampling.

The absence of [^11^C]AZ10419369 protein binding measurements is a limitation of this study, as variability in *f*_p_ could contribute to within-subject changes in *V*_T_, and may partly explain the inconsistent AZD3783 effects on *V*_T_ observed here. Extending the graphical *V*_ND_ ratio approach to the pituitary region required assuming that AZD3783 displaces [^11^C]AZ10419369 equally across the BBB. Regional variation in drug occupancy has been observed for radioligands with an affinity for more targets than the drug under investigation.^44,45^ [^11^C]AZ10419369 binds selectively to the 5-HT_1B_ receptor albeit with affinity also for the 5-HT_1D_ receptor. However, autoradiography indicates very low densities of the 5-HT_1D_ receptor in the CNS^46^ and its contribution to [^11^C]AZ10419369 binding is considered negligible.^15^ Additionally, the in vitro AZD3783 *K*_i,plasma_ closely matches the *K*_i,plasma_ estimated based on occupancy of central 5-HT_1B_ receptors^14,15^ which would be unlikely if the BBB substantially affected AZD3783 displacement of specifically bound [^11^C]AZ10419369. Given the small size and high uptake of the pituitary ROI, partial volume effects (PVE) will underestimate pituitary *V*_T_ and *BP*_ND_. However, because ROI size, and thus PVE, will be consistent within subjects, we do not expect PVE to substantially confound the analysis of pituitary [^11^C]AZ10419369 displacement by AZD3783. As partial volume correction has been known to introduce bias and amplify noise,^47^ we opted against its use in this study. The initial frame duration of 20s is longer than used in some studies to obtain estimates of blood volumes and pituitary *V*_B_ SEs exceeded those of other parameters (Supplement Table s4). However, the very high correlation between SRTM-V and SRTM-derived *BP*_ND_ (*r* = 0.94, *p* < 0.001), suggests that greater precision in *V*_B_ estimates would not alter the main finding of lack of pituitary [^11^C]AZ10419369 displacement. Finally, although our SEs indicate pituitary *V*_T_ and DVR are estimated with acceptable precision, we cannot exclude occupancy of a small population of pituitary 5-HT_1B_ receptors below detection sensitivity.

In conclusion, we could not demonstrate displacement of 5-HT_1B_ receptor radioligand [^11^C]AZ10419369 in the pituitary by the high affinity, 5-HT_1B_ receptor selective antagonist AZD3783. By plotting pituitary DVR against gray matter occupancy, we show that this is likely explained by low specific [^11^C]AZ10419369 binding in the pituitary, although the existence of a pituitary [^11^C]AZ10419369 off-target binding site not shared with AZD3783 cannot be completely ruled out. Our results highlight some of the pitfalls in estimating radioligand binding outside the brain and underscore the importance of validating non-invasive models when applied to targets suspected of having properties that differ from those of the reference region. The distinctive physiology of the pituitary, including its high vascularization and capillary permeability, may be relevant also with regards to other tracers considering that high pituitary uptake is a feature of several radioligands^12,48,49^ and poor pituitary displacement in blocking studies has been observed previously.^50,51^ Future PET studies employing plasma radioactivity corrected for protein binding as input function and autoradiography studies of pituitary tissue samples are needed to confirm low specific 5-HT_1B_ receptor binding in the pituitary region.

## Supplemental Material

sj-docx-1-jcb-10.1177_0271678X261417194 – Supplemental material for Quantification of pituitary 5-HT1B receptors with positron emission tomography: Negligible specific binding despite conspicuous uptake Supplemental material, sj-docx-1-jcb-10.1177_0271678X261417194 for Quantification of pituitary 5-HT1B receptors with positron emission tomography: Negligible specific binding despite conspicuous uptake by Martin Gärde, Katarina Varnäs, Johan Lundberg, Jonas Svensson, Lars Farde, Granville J Matheson and Mikael Tiger in Journal of Cerebral Blood Flow & Metabolism
