# Supplementary material for: Quantification of pituitary 5-HT1B receptors with positron emission tomography: Negligible specific binding despite conspicuous uptake
Source: J Cereb Blood Flow Metab. 2026 Feb 8:0271678X261417194. Online ahead of print. doi: 10.1177/0271678X261417194 (PMC12885964; doi:10.1177/0271678X261417194)
Supplement: sj-docx-1-jcb-10.1177_0271678X261417194 – Supplemental material for Quantification of pituitary 5-HT1B receptors with positron emission tomography: Negligible specific binding despite conspicuous uptake [file sj-docx-1-jcb-10.1177_0271678X261417194.docx]

Supplement

Supplementary methods

To reconstruct raw PET data, we employed standard back projection filtering with the following parameters: 128 x 128 matrix size, 2mm Hanning filter, scatter correction and a zoom factor of 2.17. The reconstructed images had a transversal resolution of 3.80 mm at FWHM while the axial resolution was 3.13 mm at the center of the field of view.

Table s1: Injected radioactivity, molar activity and injected mass at each PET

| PET | Injected radioactivity (MBq) | Molar activity (GBq/µmol) | Injected mass (µg) |
| --- | --- | --- | --- |
| Subject 1, PET 1 | 317 | 549 | 0.27 |
| Subject 1, PET 2 | 328 | 256 | 0.59 |
| Subject 1, PET 3 | 315 | 754 | 0.19 |
| Subject 1, PET 4 | 342 | 334 | 0.47 |
| Subject 2, PET 1 | 318 | 805 | 0.18 |
| Subject 2, PET 2 | 324 | 545 | 0.27 |
| Subject 2, PET 3 | 334 | 318 | 0.49 |
| Subject 2, PET 4 | 330 | 287 | 0.53 |
| Subject 3, PET 1 | 312 | 562 | 0.26 |
| Subject 3, PET 2 | 334 | 514 | 0.30 |
| Subject 3, PET 4 | 324 | 255 | 0.59 |
| Subject 4, PET 1 | 324 | 173 | 0.87 |
| Subject 4, PET 2 | 316 | 421 | 0.35 |
| Subject 4, PET 3 | 344 | 244 | 0.65 |
| Subject 4, PET 4 | 328 | 387 | 0.39 |
| Subject 5, PET 1 | 339 | 166 | 0.94 |
| Subject 5, PET 2 | 328 | 463 | 0.33 |
| Subject 5, PET 3 | 338 | 308 | 0.51 |
| Subject 5, PET 4 | 329 | 148 | 1.03 |
| Subject 6, PET 1 | 327 | 365 | 0.41 |
| Subject 6, PET 2 | 312 | 175 | 0.82 |
| Subject 7, PET 1 | 302 | 136 | 1.03 |
| Subject 8, PET 1 | 302 | 149 | 0.94 |
| Subject 9, PET 1 | 306 | 163 | 0.87 |
| Subject 10, PET 1 | 299 | 137 | 1.01 |

The injected radioactivity, molar activity and injected mass ranged between 299 – 344 MBq, 136 – 805 GBq/µmol and 0.18 – 1.03µg respectively. The largest range in injected mass within subjects was 0.33 – 1.03 (subject 5).

Table s2: Pituitary SRTM *BP*_ND_ occupancy

| Subject | Pituitary occupancy (%) at different doses of AZD3783 | | | | | |
| --- | --- | --- | --- | --- | --- | --- |
|  | 1 mg | 2 mg | 4 mg | 10 mg | 20 mg | 40 mg |
| 1 | - | -33.1 | - | 11.8 | - | 11.6 |
| 2 | - | 22.3 | - | 26.0 | - | 31.8 |
| 3 | - | -40.7 | - | -8.0 | - | NA |
| 4 | -61.2 | - | -35.2 | - | -25.1 | - |
| 5 | 10.5 | - | 13.6 | - | 20.7 | - |
| 6 | 27.2 | - | NA | - | NA | - |

Table s3: Regional brain SRTM *BP*_ND_ occupancy

| Subject | Region | Occupancy (%) at different doses of AZD3783 | | | | | |
| --- | --- | --- | --- | --- | --- | --- | --- |
|  |  | 1 mg | 2 mg | 4 mg | 10 mg | 20 mg | 40 mg |
| 1 | ACC | - | 27.6 | - | 72.0 | - | 88.2 |
|  | OFC | - | 22.4 | - | 62.7 | - | 83.5 |
|  | OC | - | 35.8 | - | 63.4 | - | 84.5 |
|  | STR | - | 23.3 | - | 70.5 | - | 88.6 |
|  | GM | - | 31.2 | - | 65.8 | - | 87.5 |
| 2 | ACC | - | 27.3 | - | 65.3 | - | 87.0 |
|  | OFC | - | 37.0 | - | 63.6 | - | 78.9 |
|  | OC | - | 26.8 | - | 55.7 | - | 73.1 |
|  | STR | - | 34.9 | - | 65.0 | - | 85.8 |
|  | GM | - | 27.9 | - | 58.7 | - | 77.1 |
| 3 | ACC | - | 37.5 | - | 43.8 | - | NA |
|  | OFC | - | 34.5 | - | 39.4 | - | NA |
|  | OC | - | 30.0 | - | 41.3 | - | NA |
|  | STR | - | 37.2 | - | 40.5 |  | NA |
|  | GM | - | 30.7 | - | 41.5 | - | NA |
| 4 | ACC | 26.5 | - | 48.2 | - | 84.6 | - |
|  | OFC | 17.4 | - | 27.5 | - | 76.3 | - |
|  | OC | 21.0 | - | 43.4 | - | 78.6 | - |
|  | STR | 21.4 | - | 43.4 | - | 87.2 | - |
|  | GM | 20.4 | - | 43.4 | - | 84.8 | - |
| 5 | ACC | 10.5 | - | 13.6 | - | 20.7 | - |
|  | OFC | 20.6 | - | 32.0 | - | 70.0 | - |
|  | OC | 19.7 | - | 31.5 | - | 60.1 | - |
|  | STR | 8.9 | - | 32.4 | - | 61.7 | - |
|  | GM | 11.2 | - | 30.1 | - | 63.4 | - |
| 6 | ACC | 27.2 | - | NA | - | NA | - |
|  | OFC | 32.3 | - | NA | - | NA | - |
|  | OC | 16.1 | - | NA | - | NA | - |
|  | STR | 45.1 | - | NA | - | NA | - |
|  | GM | 23.0 | - | NA | - | NA | - |

ACC = Anterior cingulate cortex, OFC = Orbitofrontal cortex, OC = Occipital cortex, STR = Striatum, GM = Cerebral gray matter

Table s4: Estimated standard errors (SE %) at each PET

| PET | Variable SE (%) | | | | | | |
| --- | --- | --- | --- | --- | --- | --- | --- |
|  | PIT *V*_B_ | CER *V*_B_ | PIT K1 | CER K1 | PIT *V*_T_ | CER *V*_T_ | DVR_PIT/CER_ |
| Subject 1, PET 1 | 17.84 | 7.01 | 4.38 | 1.90 | 5.58 | 2.85 | 3.11 |
| Subject 1, PET 2 | 20.86 | 10.78 | 4.42 | 3.75 | 5.13 | 4.81 | 1.89 |
| Subject 1, PET 3 | 19.30 | 15.72 | 10.21 | 12.41 | 9.78 | 11.24 | 4.19 |
| Subject 1, PET 4 | 22.58 | 3.40 | 3.76 | 1.90 | 3.31 | 2.25 | 1.47 |
| Subject 2, PET 1 | 10.53 | 7.00 | 6.80 | 8.52 | 5.21 | 7.54 | 3.31 |
| Subject 2, PET 2 | 10.80 | 6.50 | 4.28 | 5.00 | 4.35 | 6.63 | 3.04 |
| Subject 2, PET 3 | 5.16 | 3.34 | 2.22 | 2.77 | 2.21 | 5.92 | 3.95 |
| Subject 2, PET 4 | 9.11 | 5.80 | 4.71 | 4.27 | 4.45 | 5.19 | 1.94 |
| Subject 3, PET 1 | 22.34 | 8.61 | 5.99 | 9.01 | 4.92 | 7.11 | 3.11 |
| Subject 3, PET 2 | 12.58 | 11.42 | 7.43 | 21.90 | 5.78 | 15.54 | 10.38 |
| Subject 3, PET 4 | 17.76 | 8.49 | 9.56 | 13.31 | 7.00 | 9.52 | 3.96 |
| Subject 4, PET 1 | 35.10 | 6.22 | 6.59 | 2.70 | 7.43 | 4.54 | 3.62 |
| Subject 4, PET 2 | 10.54 | 4.46 | 5.99 | 4.69 | 5.79 | 8.78 | 4.01 |
| Subject 4, PET 3 | 5.33 | 3.42 | 3.54 | 3.00 | 3.68 | 6.24 | 3.13 |
| Subject 4, PET 4 | 11.13 | 4.61 | 8.65 | 9.57 | 5.83 | 11.82 | 6.75 |
| Subject 5, PET 1 | 14.17 | 7.28 | 7.29 | 7.08 | 5.03 | 4.54 | 1.85 |
| Subject 5, PET 2 | 24.01 | 8.42 | 4.87 | 3.84 | 4.94 | 4.27 | 1.84 |
| Subject 5, PET 3 | 27.78 | 4.72 | 5.37 | 3.53 | 5.09 | 4.23 | 1.94 |
| Subject 5, PET 4 | 34.11 | 16.83 | 5.92 | 6.35 | 5.73 | 5.50 | 2.11 |
| Subject 6, PET 1 | 10.22 | 6.34 | 20.33 | 14.41 | 8.94 | 7.49 | 3.39 |
| Subject 6, PET 2 | 14.62 | 4.73 | 7.82 | 9.67 | 3.52 | 6.93 | 3.89 |
| Mean | 16.95 | 7.39 | 6.92 | 7.12 | 5.45 | 6.82 | 3.47 |

PIT = Pituitary, CER = Cerebellum, DVR_PIT/CER_ = the pituitary to cerebellum distribution volume ratio

DVR_PIT/CER_ standard errors (SEs) were calculated using the following adapted^1^ equation taking into account the strong correlation between pituitary and cerebellar *V*_T_:

$$\frac{DVR SE}{\mathrm{DVR}}=\sqrt{\left( \frac{\mathrm{PIT}V_{T SE}}{\mathrm{PIT}V_{T}} \right)^{2}+\left( \frac{\mathrm{CER}V_{T SE}}{\mathrm{CER}V_{T}} \right)^{2}-2*0.93* \left( \frac{\mathrm{PIT}V_{T SE}}{\mathrm{PIT}V_{T}} \right)\left( \frac{\mathrm{CER}V_{T SE}}{\mathrm{CER}V_{T}} \right)}$$

Where 0.93 is the Pearson coefficient for pituitary and cerebellar *V*_T_. In this equation SEs refer to absolute values.

References

1. Ku HH. Notes on the use of propagation of error formulas: *NIST*, https://www.nist.gov/publications/notes-use-propagation-error-formulas (1965, accessed 8 December 2025).
